# Anti-collagenase, anti-elastase and anti-oxidant activities of extracts from 21 plants

**DOI:** 10.1186/1472-6882-9-27

**Published:** 2009-08-04

**Authors:** Tamsyn SA Thring, Pauline Hili, Declan P Naughton

**Affiliations:** 1 School of Life Sciences, Kingston University, London, KT1 2EE, UK; 2 Neal's Yard Remedies, 15 Neal's Yard, London, WC2H 9DP, UK

## Abstract

**Background:**

Owing to their roles in tissue remodelling in health and disease, several studies have reported investigations on plant extracts as inhibitors of proteinases and as anti-oxidants.

**Methods:**

The anti-ageing and anti-oxidant properties of 23 plant extracts (from 21 plant species) were assessed as anti-elastase and anti-collagenase activities and in selected anti-oxidant assays along with phenolic content.

**Results:**

Anti-elastase activities were observed for nine of the extracts with inhibitory activity in the following order: white tea (~89%), cleavers (~58%), burdock root (~51%), bladderwrack (~50%), anise and angelica (~32%). Anti-collagenase activities were exhibited by sixteen plants of which the highest activity was seen in white tea (~87%), green tea (~47%), rose tincture (~41%), and lavender (~31%). Nine plant extracts had activities against both elastase (E) and collagenase (C) and were ranked in the order of white tea (E:89%, C:87%) > bladderwrack (E:50%, C:25%) > cleavers (E:58%, C:7%) > rose tincture (E:22%, C:41%) > green tea (E:10%: C:47%) > rose aqueous (E: 24%, C:26%) > angelica (E:32%, C:17%) > anise (E:32%, C:6%) > pomegranate (E:15%, C:11%).

Total phenolic content varied between 0.05 and 0.26 mg gallic acid equivalents (GAE)/mL with the exception of white tea (0.77 mg GAE/mL). For anti-oxidant assessment, the Trolox equivalent anti-oxidant capacity (TEAC) assay revealed activity for all extracts. White tea had the highest activity equivalent to ~21 μM Trolox for a 6.25 μg aliquot. In addition, seven extracts exhibited activities = 10 μM Trolox with witch hazel (6.25 μg = 13 μM Trolox) and rose aqueous (6.25 μg = 10 μM Trolox) showing very high activities at low concentrations. A high activity for white tea was also found in the superoxide dismutase (SOD) assay in which it exhibited ~88% inhibition of reduction of nitroblue tetrazolium. High activities were also observed for green tea (86.41%), rose tincture (82.77%), witch hazel (82.05%) and rose aqueous (73.86%).

**Conclusion:**

From a panel of twenty three plant extracts, some one dozen exhibit high or satisfactory anti-collagenase or anti-elastase activities, with nine having inhibitory activity against both enzymes. These included white tea which was found to have very high phenolic content, along with high TEAC and SOD activities.

## Background

The process of skin ageing has been divided into two categories: Intrinsic and extrinsic ageing [1-3]. Intrinsic skin ageing or natural ageing is caused by changes in elasticity of the skin over time. Extrinsic skin ageing is predominately a result of exposure to solar radiation (photoageing) [1-4]. UV exposure causes physical changes to the skin due to alterations that occur in the connective tissue via the formation of lipid peroxides, cell contents and enzymes [5], and reactive oxygen species (ROS) [1,6]. Lipid peroxides can be metabolised to form secondary products which damage the extracellular matrix (ECM) while ROS are credited with involvement in the loss of skin elasticity [1,6] and in diseases such as arthritis, diabetes and cancer [6]. Biological systems need ROS for metabolic pathways and thus the body is capable of forming reactive species such as superoxide (O_2_^-^) and nitric oxide (NO) [5]. When ROS are overproduced, redox-active transition metal ions such as iron(II) or copper(II) can cause severe oxidative stress and thus damage tissues and the cellular DNA, protein, lipid and carbohydrate constituents within [6]. Superoxide dismutase (SOD) which naturally breaks down O_2_^- ^into H_2_O_2_and O_2 _has a short plasma half-life and thus novel SOD mimetics are being developed [7]. Flavonoids derived from plants can form complexes with metal ions which mean they have the potential to bind with metalloenzymes thus altering or inhibiting metabolic pathways [8] and flavonoid-metal complexes have shown potential to be SOD mimetics [9].

Eighty percent of skin dry weight is collagen which is responsible for the tensile strength of the skin. Elasticity is due to the elastin fibre network making up 2–4% of the ECM and glycoaminoglycans (GAG's) are involved in the hydration of the skin [2]. Collagen fibres, elastin fibres and GAGs are produced by fibroblasts and are primarily affected by photoageing resulting in visible changes in the skin such as wrinkles, pigmentation and changes in thickness [1,2]. ROS are also capable of inducing expression of proteinases which are responsible for remodelling the extracellular matrix such as matrix metalloproteinases (MMPs) and serine proteases [10].

MMPs are part of a group of transmembrane zinc containing endopeptidases which include collagenases and gelatinases. Collagenases are metalloproteinases capable of cleaving other molecules found within the cell for example collagenase-2 (MMP-8) can cleave aggrecan, elastin, fibronectin, gelatine and laminin as well as collagen [11]. Collagenase cleaves the X-gly bond of collagen and also synthetic peptides that contain the sequence -Pro-X-Gly-Pro where X is almost any amino acid provided that the amino terminus is blocked [12]. Collagenase from the bacteria *Clostridium histolyticum *(ChC) also degrades ECM. This bacterial collagenase hydrolyses triple-helical collagen in both physiological conditions and *in vitro *conditions using synthetic peptides as substrates [10,12]. In this study ChC was used to test the extracts for anti-collagenase activity.

Another proteolytic system involved in the degradation of the ECM is that of serine proteases one of which is elastase. Elastase, a member of the chymotrypsin family of proteases, is responsible primarily for the breakdown of elastin which is an important protein found within the ECM. Elastin, due to its unique elastic recoil properties, is vital for giving elasticity to arteries, lungs, ligaments and skin [10,13-15]. Elastases can cleave elastin as well as having a broad substrate portfolio including ability to cleave collagen, fibronectin and other ECM proteins [14,15]. As with the metalloproteinases, under normal conditions elastase activity is necessary after wounding to degrade foreign proteins within the ECM during phagocytosis by neutrophils to enable tissue repair [14,15]. In terms of anti-ageing, finding inhibitors of elastase enzymes can be useful to prevent loss of skin elasticity and thus skin sagging.

### Natural products as inhibitors of ChC and elastase

Secondary metabolites and whole extracts from plants have been widely investigated and found to have anti-collagenase and anti-elastase activities. Plants contain a wide variety of compounds including polyphenols such as flavonoids, tocopherols, phenolic acids and tannins which have been found to provide ChC inhibitory compounds or a platform on which to synthesize active molecules. Isolated green tea (*Camellia sinensis*) polyphenols such as catechin and epigallocatechin gallate (EGCG) have been found to be inhibitors of collagenase and elastase [10]. These are powerful bioflavonoids with strong anti-oxidant activity Aloe gel constituents (aloins) have also been isolated from Aloe vera (*Aloe barbadensis*) and have been found to show inhibition of collagenase *in vitro *[16]. Triterpenoids known as boswellic acids isolated from frankincense (*Boswellia *spp.) resin have also been shown to have anti-elastase activity [14]. In one study, 150 plant extracts were tested for their ability to inhibit elastase in which six plants showed activity over 65%. These included cinnamon (*Cinnamonum cassia*), turmeric (*Curcuma longa*) and nutmeg (*Myristica fragrans*) [17]. Polyphenols isolated from persimmon (*Diospyros kaki*) leaf showed anti-collagenolytic and anti-elastase activity [18]. This activity was thought to be due to the flavonoids present in the polyphenol extract. Extracts from Rosemary (*Rosmarinus officinalis*) have also been found to have good anti-elastase activity using spectrophotometric analysis [13]. Plant extracts and natural products which have shown activity in these assays represent a wide variety of the types of phenolic compounds found in higher plants.

The aim of this study was to investigate the anti-ageing and anti-oxidant properties of 23 plant extracts (Table 1) many of which are commonly found ingredients in cosmetic formulations (e.g. rose, lavender and witch hazel) [19-21]. Many intact extracts are used, along with tinctures or lyophilized powders from aqueous extractions, and thus in this study whole extracts as opposed to isolated fractions were investigated. The plants chosen are representative of 15 plant families in order to explore the diversity of polyphenolic compounds which may exhibit activity in the assays employed. The *in vitro *assays were chosen to reflect the types of damage caused by radical scavenging in skin-ageing and to find activity by plant extracts which might counteract this damage. Hence, the anti-elastase and anti-collagenase activities of the extracts are reported along with the anti-oxidant efficacies observed in the Trolox equivalent anti-oxidant capacity (TEAC) and SOD assays. The total phenolic content of each extract was also determined.

**Table 1:** Plant extracts used in this study and some of their common chemical constituents

**PLANT**	**BOTANICAL NAME**	**PLANT FAMILY**	**PART USED**	**KEY CHEMICAL CONSTITUENTS**
Alfalfa	*Medicago sativa *L.	Fabaceae	Leaf and stem	Organic acids, non-protein amino acids (canavanine), stachydrine, coumarins (medicagol), isoflavonoids (coumestrol), saponins (hederagenin) and steroids (B-sitosterol) [19].

Angelica	*Angelica archangelica *L.	Apiaceae	Root	Furanocoumarins (including xanthotoxin, angelicin, archangelin and osthol in roots) [19,20].

Anise	*Illicium verum *Hook. F.	Illiaceae	Fruit	Essential oil (up to 8% dry weight) consisting of trans-anethole, anisaldehyde, methylchavicol and other monoterpenoids [19].

Bladderwrack	*Fucus vesiculosus *L.	Fucaceae	Thallus	Alginic acid, alginates, polysaccharides and iodine [20].

Borage	*Borago officinalis *L.	Boraginaceae	Leaf, flowers and stem	Mucilages, trace amounts of pyrrolizidine alkaloids including amabiline and supinidine [19,20].

Buchu	*Agathosma betulina *(Berg) Pill.	Rutaceae	Leaf	Essential oils, mucilages, resins and flavonoids (mainly diosmin) [19].

Burdock	*Arctium lappa *L.	Asteraceae	Root	Sulfur containing Polyacetylenes in roots (including artinal and lappaphens) [19,20].

Celery	*Apium graveolens*L.	Apiaceae	Fruit	Essential oils, flavonoids, phenolic acids, coumarins and furanocoumarins [20].

Chamomile	*Matricaria recutita *L.	Asteraceae	Leaf, flowers and stem	Flavonoids (Apigenin, luteolin, patuletin-7-glycosides), coumarins (umbelliferone and herniarin) [19].

Chickweed	*Stellaria media *(L.) Vill.	Caryophyllaceae	Leaf and stem	Triterpene saponins, coumarins, phytosterols, flavonoids (apigenin, rutin), organic acids and vitamin C [19].

Cleavers	*Galium aparine *L.	Rubiaceae	Leaf and stem	Tannins, phenolic acids, flavonoids and iridoid glycosides [19].

Comfrey	*Symphytum spp*	Boraginaceae	Leaves and stem	Allantoin, mucilage, and rosmarinic acid [19].

Gotu kola	*Centella asiatica *(L.) Urb.	Apiaceae	Leaf and stem	Triterpenes (Asiatic acid and madecassic acid) and triterpenoid ester glycosides (asiaticoside and brahminoside). Also contains volatile oil [19,20].

Lavender	*Lavandula angustifolia *L.	Lamiaceae	Leaves and flowers	Essential oil monoterpenoids (including linaloyl-acetate, linalool, 1-terpinen-4-ol), leaves contain rosmarinic acid, tannins, coumarins, triterpenes and phenolic acids [19].

Mahonia	*Mahonia aquifolium *Nutt.	Berberidaceae	Fruit tincture	Roots and unripe berries contain the alkaloid berberine [19].

Milk thistle	*Silybum marianum *(L.) Gaertn.	Asteraceae	Fruit	Lipids, flavolignans (silymarin), benzodioxane (silybinin), isosilybinin, silychristin and silydianin [19].

Orange	*Citrus aurantium *subsp. *amara*	Rutaceae	Flowers	Peel contains essential oils, bitter flavonone glycosides and bitter triterpenes. Neroli oil is distilled from petals [19]. Flowers contain flavonoids (naringen and neoeriocitrin) [20].

Pomegranate	*Punica granatum *L.	Lythraceae	Glycerin fruit preparation	Fruit rind has gallotannins and ellagitannins (punicalin and punicalagen). Alkaloids present in roots, leaves, bark and young fruit but not rind [19].

Rose	*Rosa centifolia *L.	Rosaceae	Flowers (both aqueous and tincture)	Petals contain tannins, rosehips contain ascorbic acid, carotenoids, pectins, flavonoids, tannins, organic acids and sugars [19].

Tea	*Camellia sinensis *Kuntze	Theaceae	Leaf extracts of green tea (in glycerine) and white tea (lyophilized powder)	Flavan-3-ols (catechins) up to 30% dry weight, quercetin, kaempferol, other acids: gallic acid, caffeic acid, coumaric acids [22].

Witch hazel	*Hamamelis virginiana *L.	Hamamelida-ceae	Leaf	Leaves and bark both contain tannins (bark has catechols and hamamelitannins while leaves contain proanthocyanidins, ellagitannins and essential oils) [19].

Extracts indicate being diluted to 6.25 μg* and 1 μg** in TEAC assay

## Methods

### Acquisition and extraction of plants

All plant materials were acquired from Neal's Yard Remedies Ltd (Table 1). Dried herbs were ground in a pestle and mortar, extracted in boiling water at a ratio of 500 mg herb to 10 mL of boiling water and cooled prior to sonication for 15 minutes to extract maximum components from within the cells. The following day the debris was removed via filtration with Whatman no. 1 filter paper and the filtrate was passed through a 0.2 μm membrane into clean, pre-weighed glass vials. The resulting filtrates were fan dried and weighed. The dried material was stored at -20°C and re-suspended in water at 10 mg/mL for use in the assays. White tea powder was extracted in a similar manner except it was extracted in cold water and used without further processing. Pomegranate fruit and green tea leaf extracts were supplied as used in formulations in glycerine. These were dissolved in water at 10% weight by volume for use in the assays. Two tinctures (rose and mahonia in 90% ethanol) were filtered before evaporation and re-suspension in water for the assays.

### Chemicals

All chemicals were obtained from Sigma-Aldrich Ltd. (Poole, UK) unless otherwise stated.

### Collagenase assay

Prior to screening in all assays, spectra for all extracts were recorded on a Cary 300 UV-visible spectrophotometer to check for interference and shifts in the lambda max.

The assay employed was based on spectrophotometric methods reported in the literature [12] with some modifications for use in a microplate reader. The assay was performed in 50 mM Tricine buffer (pH 7.5 with 400 mM NaCl and 10 mM CaCl_2_). Collagenase from *Clostridium histolyticum *(ChC – EC.3.4.23.3) was dissolved in buffer for use at an initial concentration of 0.8 units/mL according to the supplier's activity data. The synthetic substrate *N*-[3-(2-furyl) acryloyl]-Leu-Gly-Pro-Ala (FALGPA) was dissolved in Tricine buffer to 2 mM. Plant extracts were incubated with the enzyme in buffer for 15 minutes before adding substrate to start the reaction. The final reaction mixture (150 μL total volume) contained Tricine buffer, 0.8 mM FALGPA, 0.1 units ChC and 25 μg test extracts. Negative controls were performed with water. Absorbance at 335 nm was measured immediately after adding substrate and then continuously for 20 minutes using a Cary 50 Microplate Reader in Nunc 96 well microtitre plates. EGCG, 250 μM (0.114 mg/mL) was used as a positive control.

### Elastase assay

The assay employed was based on methods from the literature [10]. This assay was performed in 0.2 mM Tris-HCL buffer (pH 8.0). Porcine pancreatic elastase (PE – E.C. 3.4.21.36), was dissolved to make a 3.33 mg/mL stock solution in sterile water. The substrate N-Succinyl-Ala-Ala-Ala-*p*-nitroanilide (AAAPVN) was dissolved in buffer at 1.6 mM. The test extracts were incubated with the enzyme for 15 minutes before adding substrate to begin the reaction. The final reaction mixture (250 μL total volume) contained buffer, 0.8 mM AAAPVN, 1 μg/mL PE and 25 μg test extract. EGCG (250 μM or 0.114 mg/mL) was used as a positive control. Negative controls were performed using water. Absorbance values between 381 and 402 nm (following pre-screen scans) were measured immediately following addition of the substrate and then continuously for 20 minutes using a Cary 50 Microplate Reader in Nunc 96 well microtitre plates.

The percentage inhibition for both of these assays is calculated by:



### Folin-Ciocalteu method

The extracts were investigated for their phenolic content using the Folin-Ciocalteu (FC) method [22]. Using 12 well Nunc plates, 100 μg (in 100 μL amounts) of the test solutions were mixed with FC reagent and then after mixing, 20% sodium carbonate was added (total volume 3.3 mL). After incubation for 2 hours measurements were recorded at 760 nm in the Cary 50 Microplate Reader. Extracts were measured using a gallic acid standard curve and equivalents were read off the straight line generated by linear regression.

### Trolox equivalent anti-oxidant assay

The anti-oxidant capacity of the plants was measured using the ABTS^+ ^(2,2'-azinobis (3-ethylbenzothiazoline-6-sulfonic acid) diammonium salt free radical assay [23]. Trolox (99.86%; Hayan Ltd, Essex, UK) standards were prepared in ethanol in the concentration range of 0–20 μM. The ABTS^+ ^free radical solution was prepared by treating 7 mM ABTS^+ ^with 2.45 mM potassium persulfate (both dissolved in PBS) to form a dark green/blue solution. This mixture was then left to stand in the dark for 16 hours. This stock solution is then diluted in PBS to give an absorbance of 0.7 at 730 nm prior to addition of 990 μL to 10 μL of Trolox (for the standard curve) and then to 10 μL of test extract (total volume of 1 mL) and measured after one minute at 730 nm on a Cary 50 MPR in Nunc 24 well plates.

### Superoxide dismutase (SOD) activity

A modified nitroblue tetrazolium (NBT) assay was employed where SOD activity was measured indirectly by producing O_2_^- ^[7]. Phosphate buffer (pH 7.8, 50 mM) was degassed under nitrogen to prepare an NBT solution containing 50 μM xanthine and 100 μM NBT. This was kept on ice in darkness for use in the assay. For the controls, 3 mL aqueous NBT solution was equilibrated to 25°C for five minutes in a Cary 300 UV-Visible spectrophotometer (thermostatically controlled) with 30 μL of distilled water. Xanthine oxidase (XO) (20 μL), diluted in buffer, was then added and the cuvette inverted to mix before being measured for three minutes at 550 nm. The enzyme concentration was then adjusted with buffer until a change in absorbance of 0.025 a.u. was achieved before testing plant extracts (10 μL extract plus 20 μL distilled water) against the control. The percentage inhibition was calculated using the rates of the control where the maximal rate is equal to 0% inhibition and 100% inhibition was where there is no absorbance change. This straight line equation can be used to extrapolate the percent inhibition of the test extracts. Superoxide dismutase (3.33 units final volume) was used as a positive control. Tests were also performed with no XO to ensure extracts did not reduce NBT (measured at 550 nm) on their own as well as without NBT to ascertain whether the extracts inhibited the formation of uric acid from xanthine by xanthine oxidase (measured at 290 nm).

## Results and discussion

### Collagenase and elastase assays

**Figure 1:**
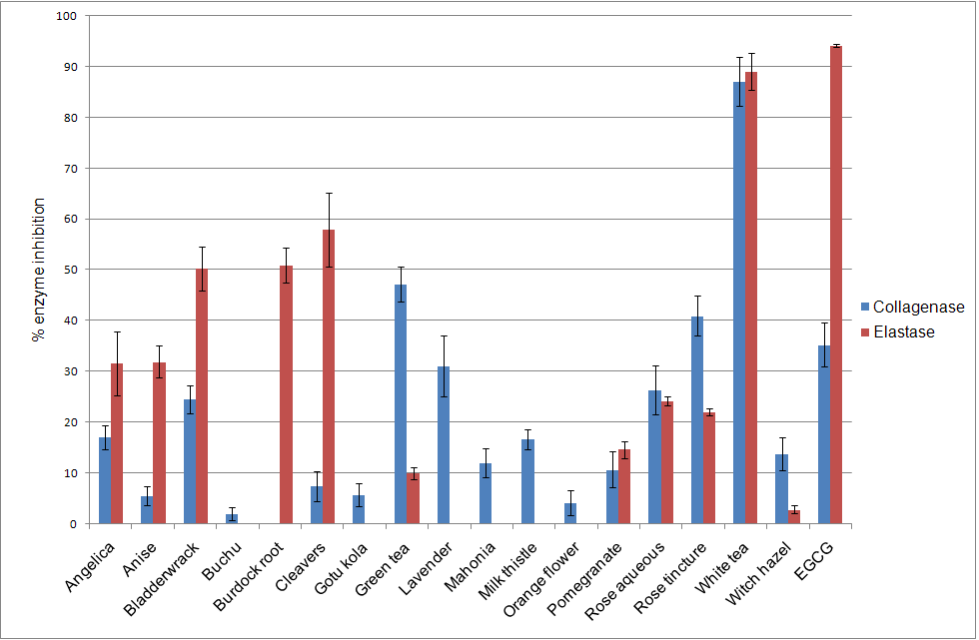
**Mean (± SEM, N = 6) inhibition of collagenase and elastase by selected plant extracts (25 μg) and EGCG**.

Preliminary screening employed the elastase and collagenase assays to rank the efficacies of the twenty three plant extracts for comparison to literature precedents. Six extracts were devoid of activity in either assay: alfalfa, borage, celery, chamomile, comfrey and stellaria. As shown in Figure 1, a wide variety of activities were exhibited by the panel of extracts against both enzymes under the conditions employed. Very high anti-elastase activities were exhibited by white tea and cleavers extracts which inhibited over 89 and 57.9% of enzyme activity respectively. In addition, good activities were exhibited by burdock root (50.9%), bladderwrack (50.2%), anise (31.9%) and angelica (31.6%). Relatively moderate anti-elastase activities were exhibited by rose aqueous (24.15%), rose tincture (22.08%), pomegranate (14.64%) and green tea (9.99%). All assay data are supplied in Additional file 1 – Appendix data table.

A similar profile was observed for the anti-collagenase activities of the selected extracts with white tea again showing the highest inhibitory activity of 87%. Furthermore, considerable anti-collagenase activities were exhibited by green tea (47.17%) and rose tincture (40.96%). Modest anti-collagenase activities (between 20–40% inhibition) were exhibited by lavender (31.06%), rose aqueous (26.39%), and bladderwrack (24.52%).

**Figure 2:**
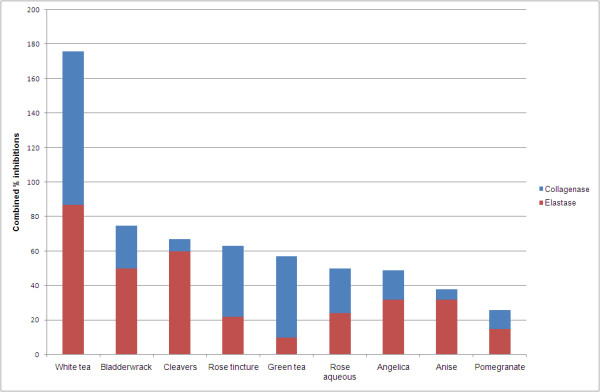
**Combined percent inhibitions of the 9 extracts found to have activity in both collagenase and elastase assays**.

It is notable that nine extracts exhibited activities in both assays (Figure 2). Expressed as aggregate activities inhibiting both enzymes, they are ranked by total % combined activities as white tea (E:89%, C:87%) > bladderwrack (E:50%, C:25%) > cleavers (E:58%, C:7%) > rose tincture (E:22%, C:41%) > green tea (E:10%: C:47%) > rose aqueous (E:24%, C:26%) > angelica (E: 32%, C:17%) > anise (E:32%, C:6%) > pomegranate (E:15%, C:11%).

Individual green tea catechins such as EGCG have already been shown to be effective protease inhibitors in the literature [10] as well as in this data set with EGCG having particularly good anti-elastase activity at 250 μM. White tea whole extract exhibits comparable anti-elastase activity to EGCG alongside very high collagenase inhibition at a very small final concentration of 25 μg which suggests additive or synergistic activity between the catechins within the tea extract particularly in the case of collagenase inhibition. Also, as collagenase is a zinc-containing metalloproteinase, the catechins within the tea extract which are known to be metal chelators may bind to the Zn^2+ ^ion within the enzyme thus preventing it from binding with the substrate [10]. In the case of the green tea extract used here, there is relatively low activity compared to that of the white tea which may be due to the extract supplied in glycerine as opposed to a pure aqueous extraction.

In a previous study, 150 methanolic plant extracts were tested against porcine and human elastases and only six extracts showed inhibition of 65% or above [17]. These extracts also only showed activity at IC_50 _values over 208 μg/mL. In this study, aqueous extracts such as white tea and cleavers exhibited good activity (89% and 58% respectively) at 25 μg final concentration for the same concentration of substrate and units/mL enzyme [17]. The afore mentioned study also tested anise methanol extracts which demonstrated anti-elastase activities of 27% at 100 μg/mL and 63% at 1000 μg/mL. In this study, aqueous anise fruit extracts were screened and a 32% inhibition at 25 μg final concentration was observed. The addition of aqueous extracts to products would be beneficial over methanolic extractions as addition of compounds extracted in methanol would require more processing whereas aqueous extracts have the potential to be added directly.

### Folin-Ciocalteu assay

**Figure 3:**
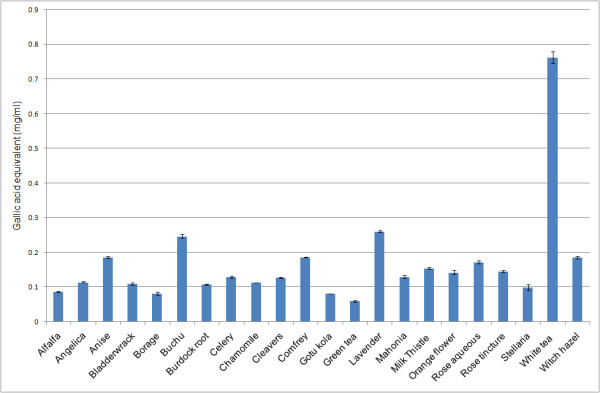
**Mean total phenolic content (shown in gallic acid equivalents) of plant extracts screened at 100 μg (± SEM, N = 3)**.

Total phenolic contents, assessed as equivalents of gallic acid, are shown for the 23 extracts in Figure 3. As expected all extracts with the exception of pomegranate contained phenolics, with the highest levels observed for the white tea (0.762 mg/mL) followed by lavender (0.261 mg/mL), buchu (0.246 mg/mL), comfrey (0.186 mg/mL), anise (0.186 mg/mL) and witch hazel (0.185 mg/mL). All other extracts exhibited gallic acid equivalents of ca. 0.1 mg/mL. It has been shown that the phenolic content and anti-oxidant capacity data can be correlated [18]. However, phenolic content is not necessarily responsible for activity as shown in this study where an extract such as pomegranate shows no phenolic content yet still exhibits mild inhibitory activity in the enzyme assays and scavenging activity in the TEAC assay.

### Trolox equivalent anti-oxidant capacity (TEAC) assay

**Figure 4:**
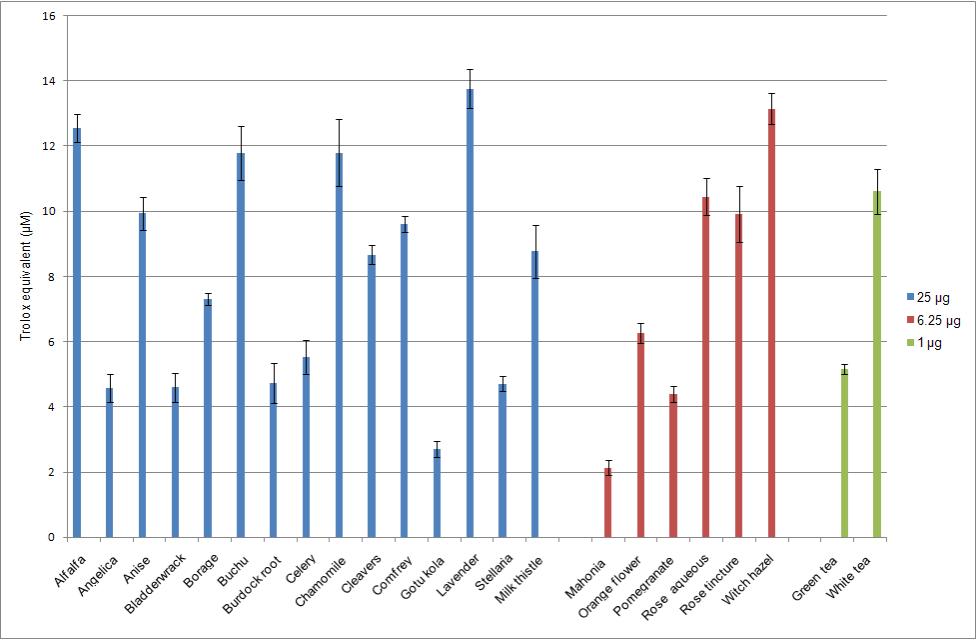
**Mean trolox equivalents of extracts at various concentrations (μg) (± SEM, N = 6)**.

It is notable that all extracts exhibited anti-oxidant activities in this assay (Figure 4). Ten extracts exhibited TEAC equivalents of ≥ 10 μM Trolox. Extracts were initially screened at 25 μg but this was too concentrated for some extracts which were then diluted further. This applied to green tea extract which was diluted to 1 μg to give the equivalent of 5.16 μM Trolox. White tea exhibited excellent TEAC activity of 10.6 μM Trolox using just 1 μg of extract.

The activities for 6 extracts were measured at 6.25 μg in the case of witch hazel (13.15 μM Trolox), rose aqueous (10.45 μM Trolox), rose tincture (9.91 μM Trolox), orange flowers (6.27 μM Trolox), pomegranate (4.40 μM Trolox) and mahonia tincture (2.13 μM Trolox).

For 25 μg aliquots of the extracts, the order of activity was lavender (13.77 μM Trolox), alfalfa (12.57 μM Trolox), chamomile (11.8 μM Trolox), buchu (11.8 μM Trolox), anise (9.94 μM Trolox), comfrey (9.61 μM Trolox), milk thistle (8.77 μM Trolox), cleavers (8.66 μM Trolox), borage (7.31 μM Trolox), celery (5.53 μM Trolox), burdock root (4.73 μM Trolox), stellaria (4.70 μM Trolox), bladderwrack (4.59 μM Trolox), angelica (4.57 μM Trolox) and gotu kola (2.7 μM Trolox). Correlation analysis (Table 2) showed a significant correlation (p = 0.001) between the total phenolic content and the TEAC values, however this was excluding white tea which was removed from the analysis due to having such considerable activity in the TEAC and having a high gallic acid equivalent.

**Table 2:** Correlation analysis Results from Pearsons correlation test

**Assays**	**P value**	**Level**
Collagenase and Elastase	0.004**	0.01 (2-tailed)

Collagenase and Total Phenolic Content (TPC)	0.000**	0.01 (2-tailed)

Elastase and TPC	0.001**	0.01 (2-tailed)

TEAC and TPC^1^	0.004**	0.01 (2-tailed)

SOD and TPC	0.045*	0.05 (2-tailed)

^1^High, outlying values for white tea are removed from the analysis

### Superoxide dismutase assay

**Figure 5:**
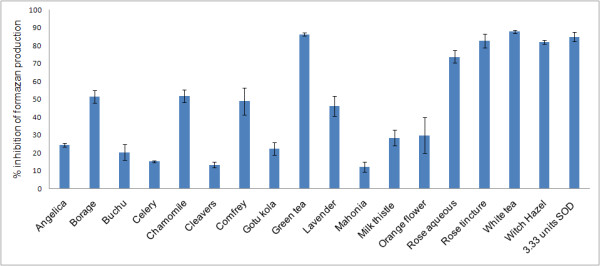
**Mean SOD activities of plants at 8.3 μg/mL compared to a Superoxide dismutase control (± SEM, N = 3)**.

Out of the 23 extracts tested for SOD mimetic activity, 17 extracts demonstrated activity (Figure 5). Six extracts showed almost no activity at all (<5%): alfalfa, anise, bladderwrack, burdock root, pomegranate and stellaria. Five extracts showed good activity (> 70%). These were the white tea (87.92%), green tea (86.41%), rose tincture (82.77%), witch hazel (82.05%) and rose aqueous (73.86%) which reflect the top performing extracts in the collagenase and TEAC assays. The tea extracts show slightly better inhibition than the SOD positive control which inhibited 85.02% of the reaction. Activity between 40 and 60% was observed for chamomile (51.94%), borage (51.56%), comfrey (48.98%) and lavender (46.31%). Five extracts showed activity between 20 and 30%: orange flower (29.89%), milk thistle (28.40%), angelica (24.48%), gotu kola (22.34%) and buchu (20.49%). Negligible activity was seen for celery (15.26%), cleavers (13.44%) and mahonia (12.24%). As mentioned previously SOD is a naturally occurring enzyme which protects the cell from the reactive and damaging O_2_^- ^by dismuting it into O_2 _and H_2_O_2 _[7] which suggests that natural products which can inhibit these enzymes *in vitro *in small amounts could have the potential to prevent radical related diseases and also skin ageing. None of the extracts showed any interference in the assay either by reducing NBT or inhibiting the formation of uric acid by XO in the presence of xanthine (data not shown). Thus the results suggest the extracts are acting in a similar manner to SOD by inhibiting formazan production directly. Correlation between SOD activity and total phenolic content was significant (Table 2).

## Conclusion

This study reveals that from a panel of 23 plant extracts, ten exhibit anti-elastase activities which are generally high or satisfactory and range up to levels of 90% inhibition. Twelve extracts exhibit high or satisfactory anti-collagenase activities ranging up to 75% inhibition. Six extracts had inhibitory activity against both enzymes. These included white tea and rose tincture which were found to have very high phenolic contents and had good scavenging activity in the TEAC assay and against superoxide radicals. The gallic acid assay shows high phenolic content for some plants for example buchu but low or no activity in the other assays.

## Competing interests

PH is an employee of Neal's Yard Remedies Ltd, which in part funded the studentship for TT.

## Authors' contributions

TT, PH, and DPN participated in the design of the study data analyses and manuscript preparation. TT, conducted the assays and analyses. All authors read and approved the final manuscript.

## Pre-publication history

The pre-publication history for this paper can be accessed here:



## Supplementary Material

Additional file 1 **Appendix data table**. Table showing numerical data values for all assays performed. Click here for file
