# Supplementary material for: Anti-collagenase, anti-elastase and anti-oxidant activities of extracts from 21 plants
Source: BMC Complement Altern Med. 2009 Aug 4;9:27. doi: 10.1186/1472-6882-9-27 (PMC2728709; doi:10.1186/1472-6882-9-27)
Supplement: Additional file 1 — Appendix data table. Table showing numerical data values for all assays performed. [file 1472-6882-9-27-S1.doc]

| **Extract** | **Gallic Acid equivalent (mg/ml)** | **± SEM** | **Trolox equivalent (µM)** | **± SEM** | **Collagenase assay**  **(% inhibition)** | **± SEM** | **Elastase assay (% inhibition)** | **± SEM** | **SOD assay**  **(% inhibition)** | **± SEM** |
| --- | --- | --- | --- | --- | --- | --- | --- | --- | --- | --- |
| Alfalfa | 0.09 | 0.002 | 12.57 | 0.43 | 0.00 | 0.00 | 0.00 | 0.00 | 0 | 0 |
| Angelica | 0.11 | 0.002 | 4.57 | 0.43 | 17.04 | 2.41 | 31.60 | 6.24 | 24.48 | 0.97 |
| Anise | 0.19 | 0.003 | 9.94 | 0.51 | 5.57 | 1.87 | 31.90 | 3.16 | 0 | 0 |
| Bladderwrack | 0.11 | 0.003 | 4.59 | 0.43 | 24.53 | 2.70 | 50.20 | 4.37 | 0 | 0 |
| Borage | 0.08 | 0.004 | 7.31 | 0.19 | 0 | 0 | 0.00 | 0.00 | 51.56 | 3.54 |
| Buchu | 0.25 | 0.01 | 11.80 | 0.82 | 1.95 | 1.30 | 0.00 | 0.00 | 20.49 | 4.44 |
| Burdock root | 0.11 | 0.002 | 4.73 | 0.61 | 0.00 | 0.00 | 50.90 | 3.49 | 0 | 0 |
| Celery | 0.13 | 0.003 | 5.53 | 0.52 | 0.00 | 0.00 | 0.00 | 0.00 | 15.26 | 0.50 |
| Chamomile | 0.11 | 0.00 | 11.80 | 1.04 | 0.00 | 0.00 | 0.00 | 0.00 | 51.94 | 3.50 |
| Cleavers | 0.13 | 0.003 | 8.66 | 0.29 | 7.39 | 3.02 | 57.90 | 7.31 | 13.44 | 1.49 |
| Comfrey | 0.19 | 0.001 | 9.61 | 0.25 | 0.00 | 0.00 | 0.00 | 0.00 | 48.98 | 7.52 |
| Gotu kola | 0.08 | 0.001 | 2.70 | 0.24 | 5.73 | 2.18 | 0.00 | 0.00 | 22.34 | 3.49 |
| Green tea** | 0.06 | 0.003 | 5.16 | 0.16 | 47.17 | 3.43 | 9.99 | 1.14 | 86.41 | 0.97 |
| Lavender | 0.26 | 0.000 | 13.77 | 0.60 | 31.06 | 6.01 | 0.00 | 0.00 | 46.31 | 5.67 |
| Mahonia* | 0.13 | 0.004 | 2.13 | 0.22 | 12.00 | 2.94 | 0.00 | 0.00 | 12.24 | 2.77 |
| Milk thistle | 0.15 | 0.002 | 8.77 | 0.81 | 16.63 | 1.91 | 0.00 | 0.00 | 28.40 | 4.34 |
| Orange flower* | 0.14 | 0.01 | 6.27 | 0.32 | 4.13 | 2.48 | 0.00 | 0.00 | 29.89 | 9.99 |
| Pomegranate* | 0.00 | 0.00 | 4.40 | 0.25 | 10.65 | 3.54 | 14.64 | 1.66 | 0 | 0 |
| Rose aqueous* | 0.17 | 0.004 | 10.46 | 0.57 | 26.39 | 4.76 | 24.15 | 0.87 | 73.86 | 3.45 |
| Rose tincture* | 0.15 | 0.003 | 9.91 | 0.85 | 40.96 | 3.90 | 22.08 | 0.64 | 82.77 | 3.85 |
| Stellaria | 0.10 | 0.017 | 4.70 | 0.23 | 0.00 | 0.00 | 0.00 | 0.00 | 0 | 0 |
| White tea ** | 0.76 | 0.017 | 10.62 | 0.69 | 87.08 | 4.79 | 89.00 | 3.62 | 87.92 | 0.93 |
| Witch hazel* | 0.19 | 0.003 | 13.15 | 0.48 | 13.70 | 3.27 | 2.80 | 0.77 | 82.05 | 1.16 |
| EGCG +ve control (0.114 mg/ml) | / | / | / | / | 35.22 | 4.33 | 94.19 | 0.33 |  |  |
| SOD control (3.33 units) | / | / | / | / |  | / | / | / | 85.02 | 2.54 |
